# Pain in Older Adults With Dementia: A Survey in Spain

**DOI:** 10.3389/fneur.2020.592366

**Published:** 2020-11-20

**Authors:** Lydia Giménez-Llort, Maria Luisa Bernal, Rachael Docking, Aida Muntsant-Soria, Virginia Torres-Lista, Antoni Bulbena, Patricia A. Schofield

**Affiliations:** ^1^Department of Psychiatry and Forensic Medicine, Universitat Autònoma de Barcelona, Cerdanyola del Vallès, Spain; ^2^Institut de Neurociències, Universitat Autònoma de Barcelona, Cerdanyola del Vallès, Spain; ^3^Department of Pharmacology and Physiology, University of Zaragoza, Zaragoza, Spain; ^4^Instituto de Investigación Sanitaria de Aragón, Zaragoza, Spain; ^5^Abertay University, Dundee, United Kingdom; ^6^Sheffield Hallam University, Sheffield, United Kingdom

**Keywords:** pain, dementia, elderly, pain assessment, pain management, International “IR” framework, guidelines, impaired cognition

## Abstract

The risk of suffering pain increases significantly throughout life, reaching the highest levels in its latest years. Prevalence of pain in nursing homes is estimated to range from 40 to 80% of residents, most of them old adults affected with dementia. It is already known that pain is under-diagnosed and under-treated in patients with severe cognitive impairment and poor/absent verbal communication, resulting in a serious impact on their quality of life, psychosocial, and physical functioning. Under-treated pain is commonly the cause of behavioral symptoms, which can lead to misuse of antipsychotic treatments. Here, we present two Regional and National Surveys in Spain (2015–2017) on the current practices, use of observational tools for pain assessment, guidelines, and policies. Results, discussed as compared to the survey across central/north Europe, confirm the professional concerns on pain in severe dementia, due to poor standardization and lack of guidelines/recommendations. In Spain, observational tools are scarcely used because of their difficulty and low reliability in severe dementia, since the poor/absent verbal communication and comprehension are considered limiting factors. Behavioral observation tools should be used while attending the patients, in a situation including rest and movement, should be short (3–5 min) and scored using a numeric scale. Among the pain items to score, “Facial expression” and “Verbalization” were considered essential and very useful, respectively. This was in contrast to “Body movements” and “Vocalizations,” respectively, according to the survey in central/north Europe. Scarce time availability for pain assessment and monitoring, together with low feasible and time-consuming tools, can make pain assessment a challenge. The presence of confounding factors, the low awareness and poor knowledge/education of specific tools for this population are worrisome. These complaints draw future directions to improve pain assessment. More time available, awareness, and involvement of the teams would also benefit pain assessment and management in cognitive impairment. The experiences and opinions recorded in these surveys in Spain and other E.U. countries were considered sources of knowledge for designing the “PAIC-15 scale,” a new internationally agreed-on meta-tool for Pain Assessment in Impaired Cognition and the “Observational pain assessment” in older persons with dementia.

## Introduction

Pain is, in all cases, a threat to human dignity, and when it comes to avoidable pain in those who cannot properly think or speak for themselves, we find ourselves faced with an imperative to join efforts to improve the situation. The risk of suffering pain increases significantly throughout life, reaching the highest levels in its latest years. It is estimated that pain exists in 50% of community-dwelling older adults, while the prevalence in nursing homes is estimated to range from 40 to 80% of residents (1). These rates indicate a serious impact on the quality of life and psychosocial and physical functioning. Although pain is a frequent complaint in residents at nursing homes, 25% do not regularly receive pain-relieving drugs (2). The administration and prescription of pain treatment occur below the experts' recommendations (3). In the older population, factors of pain management that can explain this situation are five. First, less self-report of pain. Second, pain is often present along with multiple problems and comorbidities that complicate evaluation and treatment. Third, increased incidence of side effects. Fourth, a greater potential for adverse effects and complications secondary to treatment procedures. Finally, older adults and health professionals often assume that pain is part of aging.

While pain in older adults appears to be a challenging problem for many health care professionals, it seems that pain in older adults with dementia is even more so (4). Over 50–60% of the older population living in care homes in developed countries are affected by dementia (5), while the impact of dementia in low-to-middle income countries should not be underestimated [i.e., (6)]. About 50% of the 35 million people with dementia worldwide experience pain on a regular basis. The global burden associated with the aging population projected to happen in the coming decades demands efforts to counteract its socio-economic impact but, mostly, on the quality of life of patients, caregivers and families (7).

In fact, there is no evidence to suggest that older adults with dementia experience less pain than do those without dementia (8). Thus, pain in dementia patients is assumed to be at least as prevalent as in cognitively healthy individuals of the same age. The impact that dementia and other types of cognitive damage have on the perception and expression of pain has been scarcely studied, except in Alzheimer's disease (9, 10). In this common form of dementia, extensive evidence indicates that pain sensitivity is not only intact but may even be increased. In vascular dementia, the prevalence of pain may be even higher due to a promotion of neuropathic pain (11). However, the latest studies indicate that pain is underdiagnosed and under-treated in people with cognitive damage, especially dementia (12, 13). Thus, dementia patients receive even less pain treatment than individuals without cognitive impairment, as observed in many studies. The exact reasons for this poor treatment of dementia patients have yet to be determined (14). One of the most probable reasons for under detection of pain is because, nowadays, the diagnostic tools of pain, their classification, and evaluation, depend largely on self-reports that require the intact cognitive and communication skills. Both abilities are lost throughout the disease until they are completely absent in the more advanced stages. In this scenario, it is important to note that agitation, shouting, aggression, and increased irritability are common ways for patients with dementia to express discomfort and pain. If the latter is the case, the use of antipsychotics to manage the expression of these neuropsychiatric (NPS) or behavioral (BPSD) symptoms could be inappropriate. Atypical antipsychotics are associated with a significantly greater mortality risk than placebo (15). Increased mortality risk has also been described in cerebrovascular adverse events in elderly users of antipsychotics (16). Therefore, improvement in pain assessment in the elderly demented patients is important for the proper pharmacological pain management in this population (17, 18). Currently, the use of antipsychotics and opioids and in elderly with dementia to treat BPSD is an important topic in the pharmacological management of Alzheimer's disease (19).

In this regard, a series of specialized tools have been developed since the early 1990s to assess pain in community and nursing homes, particularly in patients with communication difficulties especially dementia (20). The pros and cons of these scales are already well known in the forums on this topic. However, due to the lack of internationally coordinated research to validate these scales and select the best available solutions, almost every hospital/clinic and each research center favors its scale. At best, the consistent practice has been developed at the national level and is only described in national guidelines (21). Since age is the main risk factor for dementia and pain, it is expected that the number of patients with dementia and so much pain will also grow. These combined circumstances are of great socio-health relevance since when dementia and pain concur, their individual and social impact multiply and require transnational solutions. It is not just that there is already evidence that pain is poorly treated in dementia. Other questions, which also require an urgent response, are those regarding neurophysiological aspects of pain in dementia and pain management throughout the disease, including end-of-life dementia stages. For now, the lack of validated pain assessment tools in older people with cognitive damage, especially dementia, has thus far impeded important advances.

The COST (European Cooperation in Science and Technology) Action TD1005 “Pain Assessment in Patients with Impaired Cognition, especially Dementia” led by Stephan Lautenbacher was established to address the issues related to pain assessment and dementia [(20), see also Table 1]. The major aim was to develop PAIC, a comprehensive and internationally agreed-on assessment toolkit for older adults targeting the various subtypes of dementia and various aspects of pain, including pain diagnostics, cognitive examination and guidelines for proper assessment. In this context, Working Group 2 (WG2) developed a survey that was designed to explore the existing use of pain assessment tools and guidelines and develop an understanding of the practical considerations required to facilitate their use within clinical settings. This survey was conducted across the central European participating countries, including questions about participants' knowledge and use of existing pain assessment guidelines, the usage of existing pain assessment (observational tools) and the experience health care professionals have of implementing the tools. Recently, this survey results in central-north Europe have been reported (22), and here we present the results from southern Europe (Spain). The preliminary analysis on one of the “open questions” regarding the professional concerns on problems experienced in Spain and solutions for its improvement was presented for discussion with the scientific community of the International Psychogeriatric Association in our last IPA International Congress (23). In the present work, the results of that question have been further analyzed.

**Table 1:** State-of-art of challenges in pain assessment and management in the elderly and people with cognitive impairment, especially dementia.

**Challenges pain management in aging and cognitive impairment**
Conditionants of pain management in the elderly 1. Worse **self-report** 2. Pain is **comorbid** to pluripathology and comorbidities that complicate the evaluation and treatment 3. More incidence of **side effects** + more potential for **adverse effects** and **iatrogenesis** due to the procedure of treatments
High prevalence, needs and challenges pain diagnosis in cognitive impairment 1. **Epidemiology**: 80% in nursing homes, 50% at home are in pain 2. Cognitive decline associated to aging/MCI/Neurodegeneration 3. Pain = **Discomfort + Burden** physical-phychological-social levels 4. Infra-diagnosed 5. Infra-treated 6. Loss of **verbal communication** 7. Equal detection – pathology and stage of dementia influence the perception of pain and the pain related behavior 8. Higher tolerance 9. **NPS/BPSD** – pain manifests as agitation, shouting, aggression 10. **Scales** proxi **behavior of pain**: possible presence/absence, intensity, behavior frequency, six behavioral dimensions **FACS, vocalization, body movements, behavioral changes**, physiological changes, physical changes

*Key words are indicated in bold. NPS/BPSD, Neuropsychiatric symptoms/Behavioral and Psychological Symptoms associated to Dementia; FACS, Facial Action Coding System*.

Therefore, the aims of the present study were three. First, to conduct a preliminary survey in Spain to estimate the implementation and use of pain scoring systems in old patients with dementia and their reliability. Second, to survey on the use of standards and guidelines for pain assessment in people with cognitive impairment/dementia in Spain and, particularly, of the use of behavioral assessment scales. Finally, to obtain a detailed analysis of the current problems that health/care staff encounter and, learning from their experience and opinion, what would help improve their management.

## Methods

In the Preliminary Regional Survey, a short online questionnaire consisting of the “General information about professionals and patients” of the PAIC study was sent to an opportunistic sample of healthcare professionals (nurses and physicians) from hospitals, nursing homes, and daycare centers in Catalonia, Aragón and Comunitat Valenciana, three neighboring Spanish autonomous communities.

After this preliminary survey, the self-administered “Questionnaire about the use of standards/guidelines, instruments and professional's profile” set up by the members of WG2 of the COST Action TD1005 (22) was translated into Spanish using a backward, forward procedure. Minor modifications were necessary to clarify aspects of the different levels of qualifications and healthcare settings.

The WG2 developed the survey questions in English. The survey's focus was to explore practitioners' current use and opinions on the usefulness and usability of existing tools to identify attitudes toward assessment tools and possible barriers to their implementation.

Together with sociodemographic data, the survey included both open-ended and multiple-choice questions about participants' knowledge, use of existing pain assessment tools, and the experience healthcare professionals have of using these tools in daily practice.

The instrument contained 36 questions. In Spain's final instrument, five more questions were added to record the level of confidence the professionals had on the scales and their ability to score pain in patients with no cognitive impairment, mild cognitive impairment or dementia, and moderate/severe cognitive impairment or dementia.

The National Survey was sent online to the sampling frame consisting of all the hospitals registered at the Spanish National Health System. An opportunistic sample of nursing homes and day care centers was also included. The survey was conducted between February 2015 and November 2017.

## Data Collection

The National Survey used a probability sample of healthcare professionals. As in the previous study (22), a sample size calculation was not performed since the study aimed to describe the currently used guidelines or observational tools for pain assessment amongst older adults with cognitive impairment. The response rate was very low, with no answers from the southern part of the country. Three rounds of submissions were performed. Besides, targeted strategies were adopted to circulate the link for the web-based questionnaire. Thus, the survey was also announced on the COST-Action TD1005 website and distributed via the newsletter of other health and mental health professional groups: Sociedad Española de Infermería Geriátrica (Spanish Society of Geriatric Nursery), Master in Psychogeriatry UAB, CORE Salut Mental de Catalunya, Germanes Hospitalaries del Sagrat Cor de Jesús de Martorell, UVaMiD Unitat de Valoració de la Memòria i la Demència a Salt - Girona, and Instituto Aragonés de Salud. Respondents were not required to enter their names in the survey, and therefore it was completed anonymously. Respondents had the opportunity to stop completion of the survey at any moment, which could result in an incomplete survey.

## Analysis

Quantitative data were analyzed using SPSS version 25, and descriptive statistics were performed. For the multiple-choice answers, valid percentages were used given the variation in the number of responses per question. The open-ended questions were analyzed using content analysis. The comments to the open-ended questions were analyzed using deductive classification and superordinate categories created with an open matrix (24). A consensual agreement was evaluated by having a peer group reviewing the data to verify the responses on the open-ended questions and categories. In case of disagreement between the two researchers, it was agreed upon to discuss the differences and seek for consensus.

## Results

### Preliminary Regional Survey

**Figure 1:**
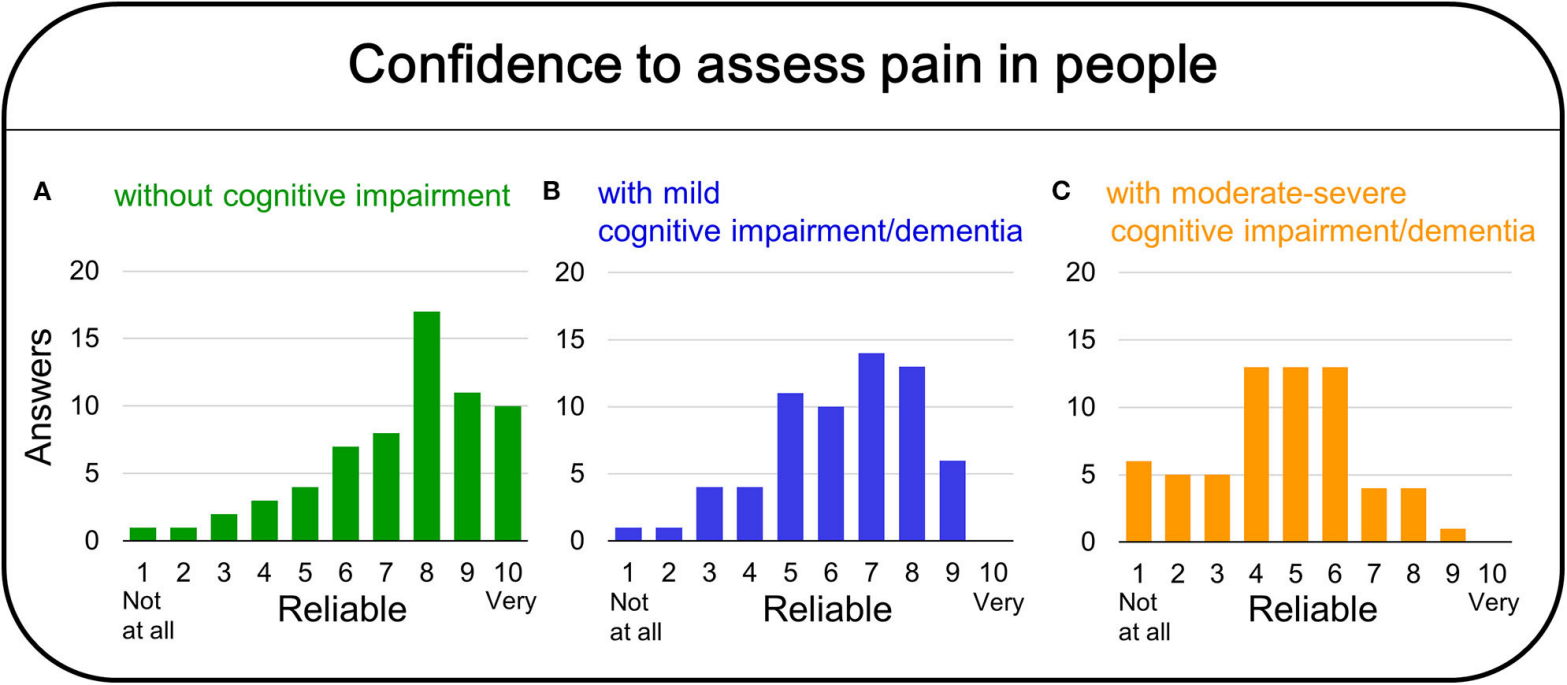
Preliminary regional survey on the confidence on pain assessment for people without cognitive impairment **(A)**, mild cognitive impairment/dementia **(B)**, and moderate/severe cognitive impairment/dementia **(C)**.

In the preliminary regional survey, 64 professionals from Catalonia, Aragón, and Comunitat Valenciana answered the questionnaire; they were mostly women +30 years old, mainly physicians and nurses. Of both sexes, their patients were mostly +60 years old, diagnosed with Alzheimer's disease, vascular, or mixed dementias and + 3Reisberg GDS stage. The duration of acquaintance with the patients was high since most of them were institutionalized patients. The 51.6% of professionals asked scored their capacity to evaluate pain between 7 and 8, with nurses self-reporting a higher score than physicians. 59.7% of professionals use pain scale (mostly EVA and observational scales), being bit more used in nursing homes than in hospitals. Forty-five percentage of professionals used the pain instruments daily or at least once per week. Overall, the reliability given to the scales was 1 or 2 ranks lower than for their own professional ability to detect pain in the patients (Figure 1).

### Participants in the National Survey

**Figure 2:**
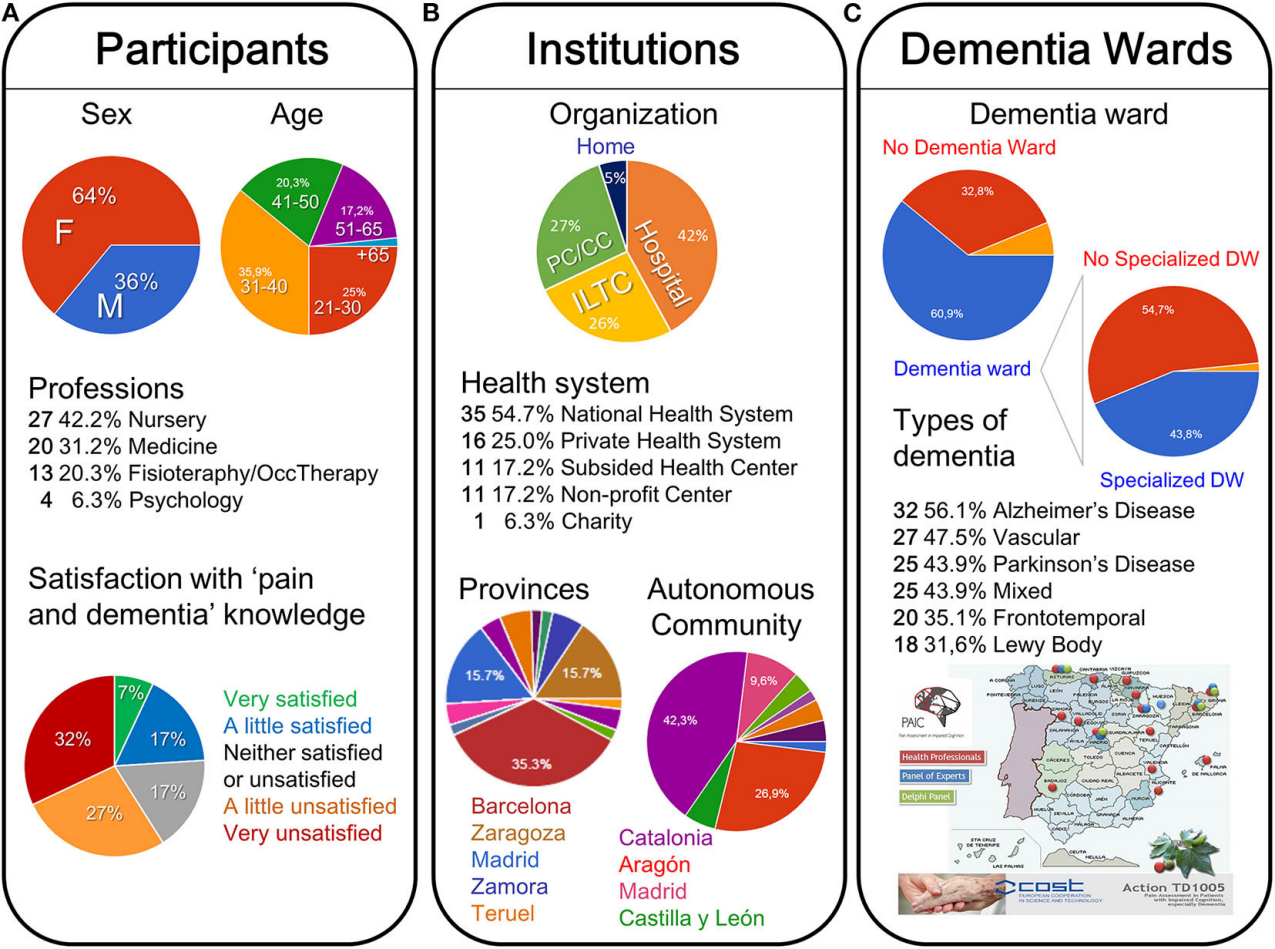
Participants **(A)**, institutions **(B)**, and dementia wards **(C)** profiles in the National Survey and geographical distribution of the answers received and panels of professionals.

The results summarize the answers of 64 Spanish health professionals working with older people with cognitive impairment (dementia). Despite respondents having the opportunity to stop completing the survey at any moment, which could result in an incomplete survey, all the submissions were completed surveys. Noticeable, nor submissions were received from the southern area of Spain in any of the three rounds. Figure 2 depicts the participants (Figure 2A), institutional (Figure 2B) and dementia wards (Figure 2C) profiles. The participant's age was normally distributed, from 21 to 65 years old, with 1/3 of the participants being 31–40 years old. Concerning their sex, the sample of participants was enriched in females (64%). There was a similar composition of registered nurses, medical practitioners, and therapeutic professions (physiotherapists, occupational therapy, psychologists). They worked in public and private hospitals, nursing homes, and day-care centers of 16 different regional areas of Spain (see map in Figure 2C). Most of the institutions had a dementia ward (60.9%), which was specialized in 43.8% of cases (Figure 2C). Several types of dementia were referred by participants as those requesting their professional attention, ranging from 56.1% of professionals taking care of Alzheimer's disease patients to 31.6% of them caring for people with Lewy body dementia.

### Pain Education

Only 14% of participants have ever received post-registration education relating to pain assessment in people with cognitive impairment. Similarly, only 19% reported that case conferences or multidisciplinary team meetings are held about managing pain in cognitive impairment. Accordingly (see Figure 2A), 58% of professionals reported feeling very (32%) or slightly (27%) dissatisfied with their knowledge about this important issue, despite their efforts to do it well, paying special attention and taking into account the family and caregivers reports. Thus, there are few opportunities for multidisciplinary team meetings or specialized pain education, mostly thanks to pain committees and online courses of pain education for health professionals. However, health professionals that received them reported high satisfaction with their knowledge and management of pain.

### Use of Standards/Guidelines

**Figure 3:**
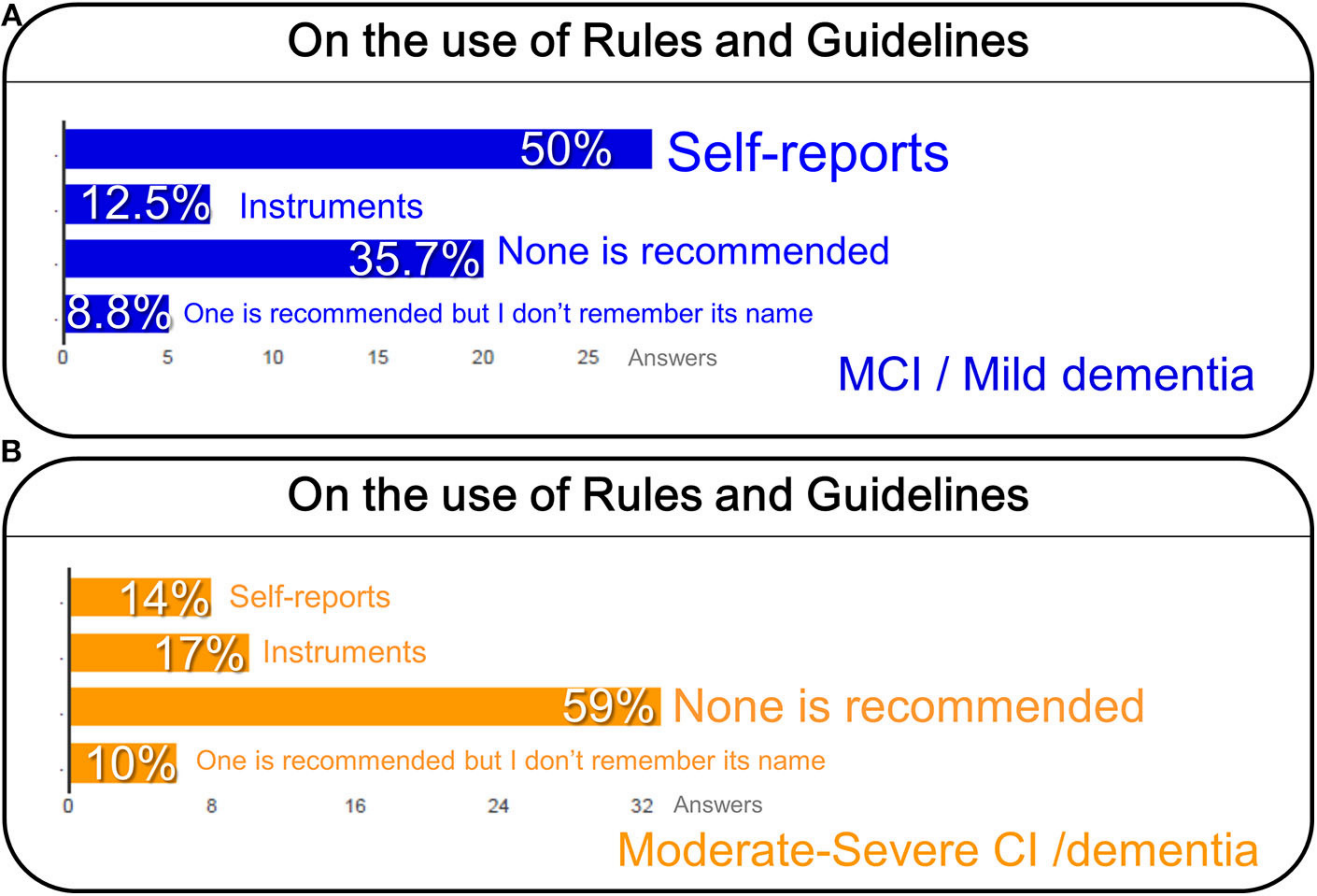
On the use of rules and guidelines for pain assessment in people with mild cognitive impairment (MCI) or mild dementia **(A)**, and cognitive impairment (CI), and severe dementia **(B)**.

On the use of rules and guidelines, self-reporting pain assessment tools were the most recommended (50%) for people able to use self-report (i.e., with mild dementia/cognitive impairment) (Figure 3A). However, a high percentage (35.7%) of their institutions do not recommend a tool (Figure 3A); this increased to 59% for those unable to use self-report (Figure 3B). Interestingly, behavioral/observational instruments (e.g., Abbey, PainAD) were poorly recommended in people with dementia with mild (12.5%, Figure 3A) to moderate/severe conditions (17%, Figure 3B).

**Figure 4:**
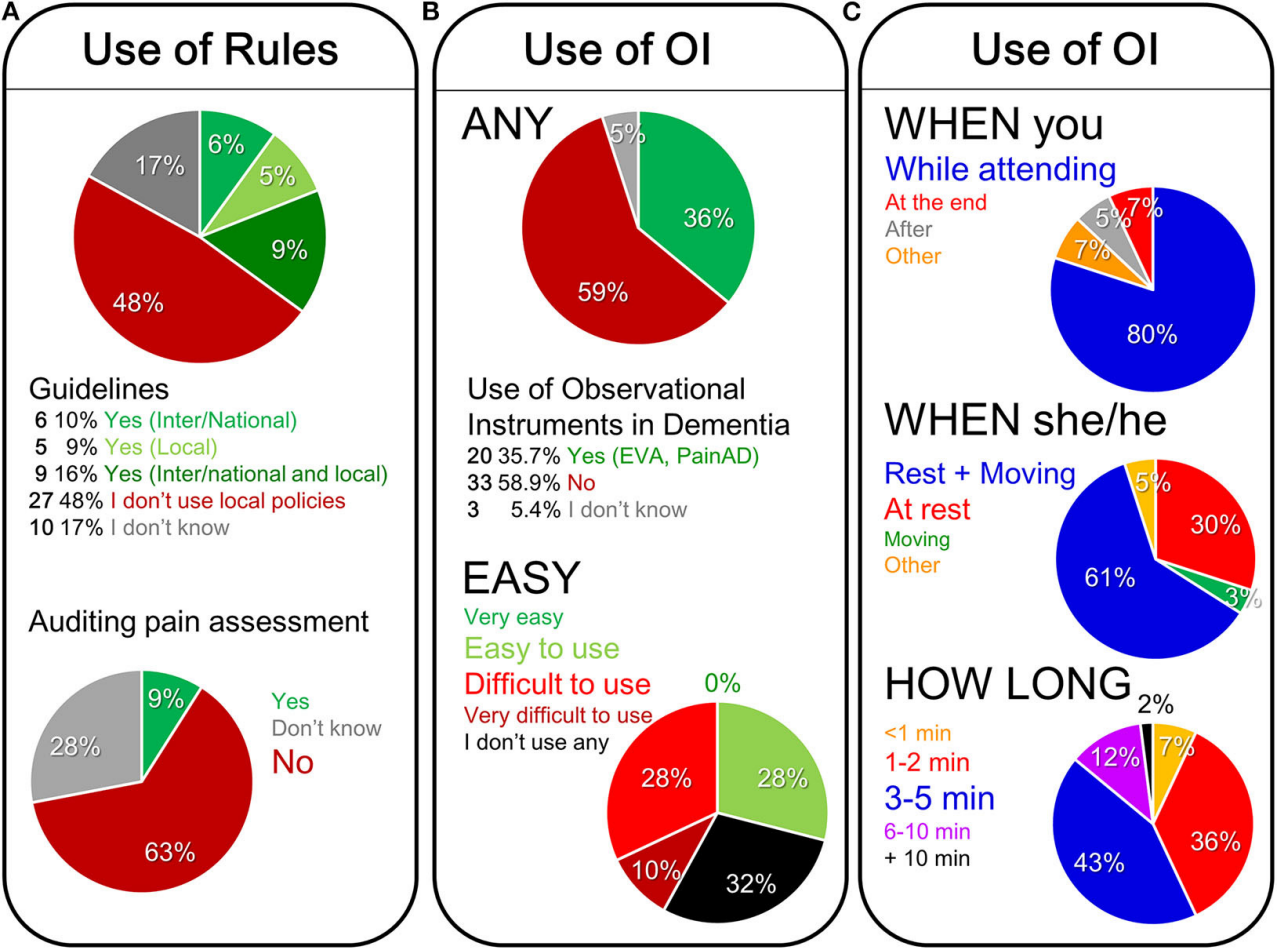
On the use of rules and audition of pain assessment **(A)**, the use of observational instruments **(B)**, and the different conditioning factors on the use of observational instruments for pain assessment in people with dementia **(C)**.

In fact, when asking about the source of recommendations, the participants informed that the use of national/international standards/guidelines in their institutions or the existence of local policies for pain assessment in people with cognitive impairment associated to dementia was poor (Figure 4A). Thus, more than half of professionals (65%) indicated no guidelines or local policy (48%) or did not know if their institution had any (17%), in contrast to that 35% of professionals that use a local policy and/or national/international guidelines (Figure 4A). The ratio of organizations reporting auditing pain assessment in older people with dementia was low, as it could only be confirmed by 9% of participants (Figure 4A).

### Use of Observational Pain Assessment Tools

As shown in Figure 4B on the use of observational instruments, only 35.7% of participants use them for pain assessment in people with dementia in the current practice. In fact, EVA, PAINAD-sp and observational scales were the most commonly used pain assessment scales reported. Overall, the participants find these tools difficult (28%) or very difficult to use (10%), they have medium to high reliability, but none of the participants gave them a 10 out of 10. Thirty-two percent do not use observational tools.

Regarding the participants opinion about the clinical settings to use of observational instruments (Figure 4C), they informed that the behavioral pain assessment is mostly (80%) conducted while caring for a patient and over a period of time involving rest and movement (61%) or at rest (30%). When asking about preferences, the professionals considered it ideal for a behavioral pain assessment to take 3–5 min (43%) or 1–2 min (36%).

**Figure 5:**
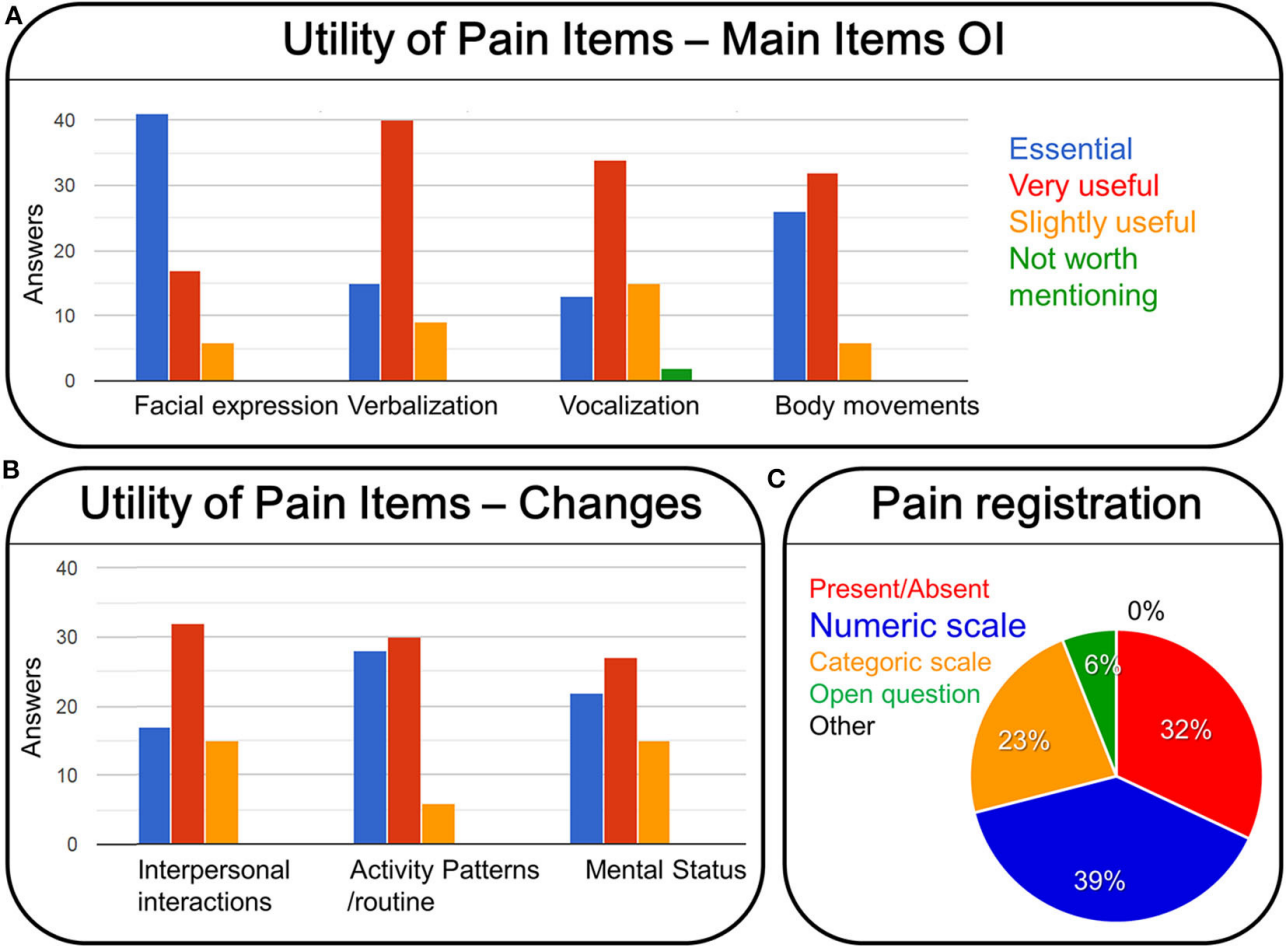
Utility of pain items of observational scales for pain assessment in people with dementia (**A**, main items: facial expression, verbalization, vocalization, body movements; **B**, changes in interpersonal interactions, activity patterns/routine, mental status), and the professional preference about how to register pain **(C)**.

Regarding the utility of the pain items in the observational instruments (Figure 5), facial expression (41%) followed by changes in activity patterns or routines (28%) were considered by professionals as essential elements of a behavioral pain assessment. Verbalizations (40%), followed by vocalizations (34%), body movements and changes in interpersonal interactions (both 32%), and changes in mental status (27%) were considered as very useful (see Figures 5A,B).

In the different institutions, pain assessment amongst people with cognitive impairment is conducted mostly by registered nurses and medical practitioners (50%). It is recorded in the patients' clinical history and “nursery working sheets,” and its mainly communicated to medical practitioners (59%). Registered nurses (43%), family members and other important persons apart from family (46%), as well as members of therapeutic professional groups (41%) are equally informed of the results of pain assessment. When using a behavioral pain assessment tool, the professionals would prefer to respond to items by rating items on a numeric scale (39%), or ticking a simple present/absent (32%) or selecting categories (i.e., slight moderate/a lot) (23%) (see Figure 5C).

### Challenges and Future Directions

**Figure 6:**
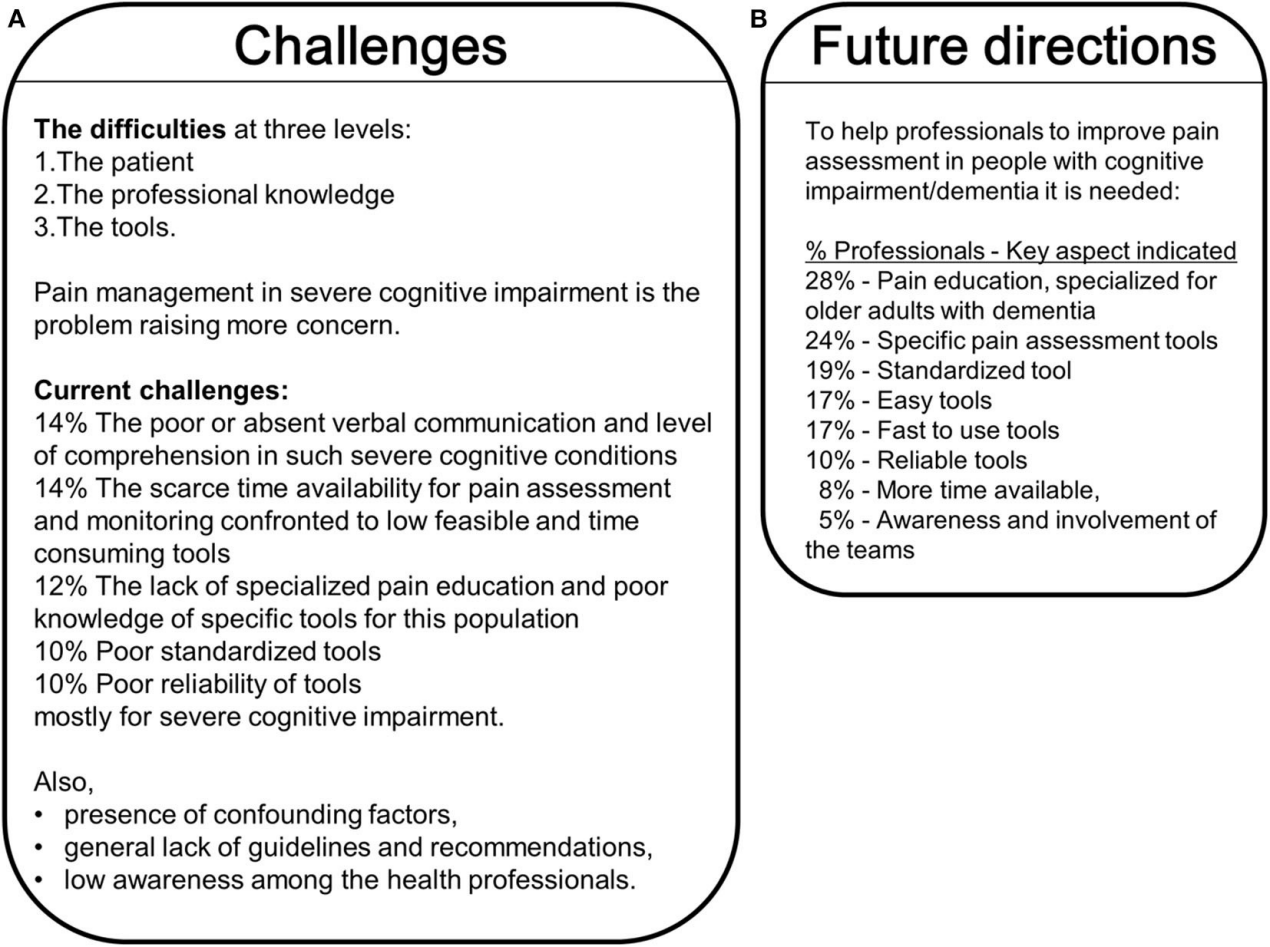
Challenges **(A)** and Future directions **(B)** in the pain assessment and management in the cognitively impaired/dementia population.

Figure 6 details the challenges reported by Spanish professionals in assessing pain in cognitive impairment (Figure 6A), and the solutions and recommendations for its improvement (Figure 6B). In an open question, there was a consensus among the different professionals that the difficulties were found at three levels: the patient, the professional knowledge and the tools. They pointed out pain management in severe cognitive impairment, due to its complexity and diverse etiology, like raising more concern. The difficulties are currently found in the poor or absent verbal communication and level of comprehension in such severe cognitive conditions (14%); the scarce time availability for pain assessment and monitoring confronted to low feasible and time-consuming tools (14%); the lack of specialized pain education and poor knowledge of specific tools for this population (12%); as well as the poor standardization (10%) and reliability (10%) of the tools, mostly for severe cognitive impairment. Also, the professionals referred to difficulties due to the presence of confounding factors, general lack of guidelines and recommendations, low awareness among the health professionals.

When the participants were asked what would help them improve pain assessment in people with cognitive impairment/dementia (Figure 6B), their answers focused on pain education specialized for this population (28%). They also stated the need for pain assessment tools to be: specific (24%), standardized (19%), easy and fast to use (17%), and reliable (10%). More time available (8%), awareness and involvement of the teams (5%) were also commonly referred to as aspects that would benefit pain management in cognitive impairment.

## Discussion/Conclusions

Pain assessment among older adults is often neglected or not done (21, 25, 26). Two-thirds of residents in long term care facilities have dementia (5). Pain identification, measurement, and management confront a series of difficulties due to the several forms of dementia, their different etiology, neurodegeneration processes, and worsening by the progressive loss of verbal communication and self-reports. Thus, these persons are more likely not to have their pain assessed and therefore undertreated (27). Untreated pain is not only distressing for the individual, but causes other problems, including reduced quality of life, interrupted or poor sleep patterns, impaired social interactions, and reduced appetite. Severe pain is less likely to cause wandering but more likely to display aggressive and agitated behaviors (28). Conversely, treating pain in patients has been reported to have concomitant relief of agitation (29). The high number of physical assaults on staff working in dementia wards (30) may be related to unidentified and unmanaged pain and often results in antipsychotic medication rather than person-centered care. In such a case, a worrisome clinical situation arises due to the increased mortality risk that has been associated with antipsychotics in the elder but mostly those with dementia (15, 16). Therefore, “Pain in Cognitive Impairment, especially Dementia” is one of the health topics with clear snail effect (poor and slow diagnosis in frail elderly patients) (31) that raises growing concern among professionals since the pain in these patients is known to be under-detected and under-treated (13, 32). Despite this evidence, statements such as “pain is a normal part of the aging process,” “the older person who has dementia cannot feel pain,” or “if an older person does not verbalize pain, it does not exist” are often displayed amongst health care professionals and result in poor pain management for these elderly groups (33, 34). Also, the belief that older adults should not be prescribed strong opioids results in avoidance of their use (35, 36).

In the present work, we estimated a low implementation and use of pain scoring systems in patients with cognitive impairment/dementia, probably to the poor reliability given to them by Spanish professionals. The survey reflected that the lack of guidelines is a major problem. Similarly, the ratio of organizations reporting auditions of pain assessment in older people with dementia to assess care quality was low. The low response rate (taking part in the study) and an important percentage of uncertain responses (I don't know) were also notorious. Most research works never achieve a 100% response rate, and reasons for low rates include refusal, ineligibility, inability to respond, and contact not been possible (37). Limitations associated with poor response are referred to as each non-response being liable to sample bias. Here, the participants found observational tools difficult or very difficult to use, an important number do not use them, and many complained they have low reliability in the advanced stages of the disease. Therefore, the current study confirmed many findings from the original E.U. survey (22). The majority of participants did not use national/international standards, guidelines, or local policies or were unsure if their institutions had any pain management guidelines (E.U. survey, 42, and 17%, respectively). In many other countries globally, pain is not routinely assessed, even though we have the tools available to help us do this.

An important number of observational scales have been developed so far to assess pain in persons with dementia, and their use seems to respond to experience-based confidence in them (20). The EU-COST-Action “Pain in impaired cognition, especially dementia” selected items out of existing observational scales, critically re-assessed their suitability to detect pain in dementia. The EU-consortium created an improved “best-of” meta-tool built on the knowledge and expertise implemented in these scales (38). In both the current study and the E.U. survey, approximately one-third reported using observational pain assessment tools for older people with dementia (36 vs. 34%). Furthermore, in both studies, most of the respondents complete the observational assessment while providing care for the patient and over a period of rest and movement, with a preference for short assessments of 3–5 min and numeric or easy tick scales, as opposed to selecting categories or open-ended questions. In the original survey, participants from the U.K. unsurprisingly demonstrated a preference for the Abbey scale, which is simple and easy to apply. In contrast, other countries in the previous study opted for the Doloplus scale. Interestingly, the Spanish survey participants did not choose the Abbey pain scale as a preferred tool.

While there were similarities in the preference for verbal, vocal, and body movements as being essential elements of a behavioral pain assessment, the Spanish participants reported facial expression as the most important and essential pain indicator (41%), the original survey found facial expressions were regarded as being less important in the clinical setting (18% rated as essential). Facial expressions were reported as not easily observed when providing care, such as washing or moving a patient. Probably, the relevance of facial expressions to infer pain depends on cultural aspects on the expressivity (how one expresses) and detection and codification (how one detects from others) of non-verbal communication, known to be higher in Latin countries. In fact, checklists of non-verbal pain indicators (39) do a specific analysis of the facial expression coding system (FACS). Most studies on the use of FACS on pain in adults and elderly health conditions, or cognitive deficits and/or chronic pain, show that it is a reliable and objective tool in the detection and quantification of pain in all patients (40, 41).

Regarding the professional concerns on problems experienced in Spain and solutions for its improvement (23), pain management in patients with severe cognitive impairment was mostly the one raising more concern. The poor standardization of practices, poor or lack of guidelines or recommendations for the complex and heterogeneous features of the last stages of dementia were also considered as limiting point for their professional activity. Here, the detailed analysis of the answers unveils the specific aspects behind these reasons. First, the main functional limitation was the poor or absent verbal communication of the patient but also of his/her comprehension. Second, the low feasible and time-consuming tools in a professional activity with a short time to assess the patients were seen as worsening pain assessment challenges, mostly in these patients with severe cognitive impairment. Third, the professionals' complain about knowledge and education indicated that the poor knowledge of specific tools for pain assessment in these populations and the lack of specialized pain education were important regrets. Standardization and reliability of tools, mostly for severe cognitive impairment, were also mentioned by many professionals, as well as confounding factors, and low awareness among health professionals. Most of these challenges pointed out by Spanish professionals agreed with aspects reported from the original survey (22). There, concerns referred to uncertainty about the observation; lack of information; lack of objectivity; lack of education, knowledge and expertise; lack of time; lack of interest and awareness; and lack of available pain tools. In the current survey, the key challenges were similar and focused on difficulties in communicating with the patient; short time available for pain assessment and monitoring; lack of specialized pain education and poor knowledge of tools suitable for this population; poor standardization and reliability of tools; and general lack of guidance and recommendations.

Inadequate pain education from both studies came out as a key problem relating to pain assessment in dementia patients. In Spain, professionals reported feeling very (32%) or slightly (26%) dissatisfied with their pain assessment knowledge. Health professionals who did have multidisciplinary staff meetings regarding pain assessment and specialized pain training reported higher satisfaction with their knowledge and pain management. This study's findings indicate that training needs are still not being met, despite the clinician's best efforts to learn.

It is evident from the comparable findings that health professionals across the E.U. struggle with the same challenges in pain assessment in people with dementia. The recommendations made by participants from Spain closely align with those made by authors of the E.U. survey; these include improved pain education for understanding pain assessment and management in people with dementia; improved pain assessment tools that are fast and easy to use and interpret; and more time allowed for pain assessment. Efforts to establish more feasible instruments and policies together with education are key targets of our future directions in Spain as they are for other countries.

Among the limitations of the study, the first to be mentioned is the number of participants, lower than in the center-north Europe survey, mostly due to the lack of submissions received from an important geographical area. The length and the number of sensitive questions regarding the use of guidelines probably was a strong limitation to receive submissions. As mentioned before, despite the chance to stop completing the survey at any moment, all the submissions were completed surveys. The sex bias of participants, mostly females, was observed but probably less than expected for the gender bias in healthcare professions. Finally, since there are different types and degrees of dementia, a survey requesting answers for each one of them would be more specific.

The Spanish survey was in clear agreement in the results obtained in center-north Europe, except for considering facial expression as a key aspect of observational tools, probably due to cultural bias. This convergence in the professional criteria through south-center-north Europe is relevant to note because, in Spain, the distribution of the survey to different health professional profiles, not always confident with the approach to patients with severe dementia, could have been a source of bias in the findings and interpretation of the results.

The results of the present work and the center-north Europe survey (22) were considered as one of the sources of knowledge for the PAIC-15 scale, a new internationally agreed-on meta-tool for Pain Assessment in Impaired Cognition, composed by a list of 15 observational items that have demonstrated psychometric quality and clinical usefulness both in their former scales and in their international evaluation (38). Also, these surveys' professional opinions were taken into consideration in the scientific development and discussion of our most recent work on observational pain assessment in older persons with dementia (42).

## Data Availability Statement

The raw data supporting the conclusions of this article will be made available by the authors, without undue reservation.

## Author Contributions

LG-L and PS: WG2 COST-Action TD1005 development of the concept and study design. LG-L, MB, AM-S, and VT-L: study conduct, survey and data collection from the different geographical areas. LG-L: data analysis and illustrations. LG-L, RD, and PS: drafting manuscript. AB and MB: critical discussion. All authors participated in the scientific discussions and approved final version of manuscript.

## Conflict of Interest

The authors declare that the research was conducted in the absence of any commercial or financial relationships that could be construed as a potential conflict of interest.
